# The RNA polymerase trigger loop functions in all three phases of the transcription cycle

**DOI:** 10.1093/nar/gkt433

**Published:** 2013-05-21

**Authors:** Thomas Fouqueau, Mirijam E. Zeller, Alan C. Cheung, Patrick Cramer, Michael Thomm

**Affiliations:** ^1^Institut of Microbiology and Archaea Center, Universität Regensburg, 93053 Regensburg, Germany and ^2^Gene Center and Department of Biochemistry, Center of Integrated Protein Science Munich (CIPSM), Ludwig-Maximilians-Universität München, 81377 Munich, Germany

## Abstract

The trigger loop (TL) forms a conserved element in the RNA polymerase active centre that functions in the elongation phase of transcription. Here, we show that the TL also functions in transcription initiation and termination. Using recombinant variants of RNA polymerase from *Pyrococcus furiosus* and a reconstituted transcription system, we demonstrate that the TL is essential for initial RNA synthesis until a complete DNA–RNA hybrid is formed. The archaeal TL is further important for transcription fidelity during nucleotide incorporation, but not for RNA cleavage during proofreading. A conserved glutamine residue in the TL binds the 2’-OH group of the nucleoside triphosphate (NTP) to discriminate NTPs from dNTPs. The TL also prevents aberrant transcription termination at non-terminator sites.

## INTRODUCTION

RNA polymerases (RNAPs) carry out transcription in all living organisms. One RNAP is present in bacteria and archaea, whereas eukaryotes possess three to five specialized nuclear RNAPs I-V. RNAP structures suggested mechanisms of transcription that can now be analysed biochemically. Analysis of eukaryotic RNAP II mutants is however restricted to viable yeast strains, but archaeal RNAP, which is closely related to RNAP II (1,2), is available in recombinant form and used for functional studies (3–9).

Archaeal RNAP and eukaryotic RNAP II use the same core promoter elements, the TATA box and TFIIB recognition element (BRE), and interact with homologous general transcription initiation factors (GTIFs), TATA-binding protein (TBP) and transcription factor B (TF(II)B), which govern promoter DNA recognition and opening (7,10–12). A third archaeal initiation factor, transcription factor E (TFE), corresponds to the N-terminal part of eukaryotic TFIIEα and interacts with the DNA non-template strand to stabilize the pre-initiation complex (3,4,7). RNAP enters abortive transcription and repeatedly synthesizes short transcripts (13,14). When the RNA reaches a critical length, RNAP dissociates from GTIFs and enters productive elongation (15,16). The RNAP active site uses two Mg^2+^ ions to catalyse RNA chain growth by phosphodiester bond formation.

The trigger loop (TL) is a conserved mobile element of the RNAP active centre (17–19). In *Pyrococcus furiosus* (*Pfu*) RNAP, the TL is part of the subunit A'', which together with subunit A' corresponds to RNAP II subunit Rpb1. Five distinct TL conformations have been observed: open, closed, wedged, trapped and locked (20). Nucleoside triphosphate (NTP) substrate binding induces a conformational change of the TL from the ‘open’ to the ‘closed’ state (19,18,21–23). After catalysis, a ‘wedged’ TL conformation may accompany translocation of nucleic acids (21,24–27). When RNAP II reverses movement, it backtracks along DNA and RNA (28), until it arrests, and this has been attributed to a ‘trapped’ TL state (29). Finally, the transcript cleavage stimulatory factor TFIIS (TFS in archaea) displaces the TL from the active site and induces a ‘locked’ conformation (29,30).

In yeast and bacteria, the closed TL contacts the NTP through two residues, Rpb1 L1081 (*P. furiosus* A'' L83, *Escherichia coli* β' M932) and H1085 (*P. furiosus* A'' H87, *E. coli* β' H936) (18,19,23). These contacts to the incoming NTP play a role during NTP selection (22,31). The leucine residue was proposed to maintain the correct position of the NTP by base-stacking interactions (32). The invariant histidine was proposed to participate in acid-base catalysis and nucleotidyl transfer by interacting with the NTP β-phosphate when the TL is closed (19,31,32). The histidine was proposed to act as a proton donor for pyrophosphate formation and release (33–35). The TL is targeted by the transcription inhibitors streptolydigin and tagetitoxin in bacteria (36–38) and by the mushroom toxin α-amanitin in yeast (21,31).

Although a role of the TL in NTP selection and catalysis is established, other aspects of TL function remain unclear, including its role in the discrimination of NTPs from dNTPs. In yeast and bacteria, an asparagine residue at the metal-binding aspartate loop (*Saccharomyces cerevisiae* Rpb1 N479, *P. furiosus* A' N456, *Thermus thermophilus* β' N737) was suggested to recognize the 2'-OH group of the NTP (19,39). The TL was proposed to contribute to discrimination of NTPs against 2'-dNTPs via its histidine residue (31). However, recent structural data showed that the yeast RNAP II TL residue Rpb1 Q1078 (*P. furiosus* A'' Q80, *E. coli* β' Q929) contacts the NTP 2'-OH group (23).

The role of the TL during RNA proofreading is also unclear. If a nucleotide is misincorporated, the mismatched 3′-nucleotide can fray from the DNA template to inhibit further RNA extension (40). After backtracking by one position, a RNA dinucleotide comprising the misincorporated nucleotide can be removed by the intrinsic endonucleolytic cleavage activity or by factor-dependent cleavage stimulation (Gre factors in bacteria, TFIIS in eukaryotes, TFS in archaea) (41–45). RNA cleavage frees the NTP site and creates a new 3′-OH group in the active site, allowing transcription to resume. In bacterial RNAP, intrinsic RNA cleavage is inhibited by streptolydigin and requires the TL (36,37,46). Another study however observed that factor-dependent RNA cleavage was TL independent (47).

The mechanism of transcription termination in archaea is related to termination by eukaryotic RNAP III because oligo-dT sequences act as a termination signal and facilitate transcription re-inititaion (48–51). Unlike in bacteria or in the eukaryotic RNAP II system, termination by archaeal RNAP is independent of RNA secondary structures upstream of the termination sequence and apparently does not require termination factors (49,50,52–55).

Here, we present a functional dissection of the TL by using the reconstituted recombinant *Pfu* RNAP transcription system (4,56). This allowed us to introduce alanine substitutions and deletion mutations and to selectively analyse the TL and its key residues. We provide the first analysis of TL function in all three phases of the transcription cycle and unravel previously unknown TL functions.

## MATERIALS AND METHODS

### Recombinant protein production

Recombinant RNAP was reconstituted as previously established (4). Recombinant transcription factors TBP, TFB TFE were produced as described (4,7).

### Promoter-dependent transcription assays

Standard transcription template *gdh*-C20 (57) was generated by PCR using M13 primers, following purification with the Qiagen PCR purification kit. The 297 bp PCR product contains the promoter region of a modified version of the *Pfu gdh-*gene, where the first cytidine in the RNA-coding strand occurs at position +20. Run-off transcription yields a 113 nt RNA product. Synthetic oligonucleotide templates were generated as described (58). Run-off transcription was carried out as described (7). Briefly, RNAP was incubated with 10 nM *gdh*-C20 template, 100 nM TBP and 70 nM TFB in a final volume of 25 μl in transcription buffer containing, NTPs (440 μM ATP, 440 μM GTP, 440 μM CTP, 2.7 μM UTP) and [α-^32^P]UTP at 0.15 MBq (110 tBq/mmol) for 15 min at 70°C. Transcription initiation was carried out with the template *gdh*-C9, where the first cytidine occurs at position +10, 40 μM priming RNA (Sigma), [α-^32^P] complement NTP at 0.22 MBq (110 tBq/mmol). RNA was extracted with phenol-chloroform and analysed on an 8% (run off) or 28% (trinucleotides and short transcripts) polyacrylamide gel containing 7 M urea.

### KMnO_4_ footprinting

Thymidine residues in open complexes were detected by treatment with potassium permanganate as previously established (57). Reconstituted RNAP was pre-incubated for 15 min at 70°C with a DNA fragment encoding the *gdh*-C20 promoter in reactions containing 90 nM TFE, 50 nM RpoE', 100 nM TBP and 70 nM TFB.

### Bead-based RNA extension and TFS-induced cleavage assays

Bead-based elongation complexes (ECs) containing complete complementary scaffolds were assembled and immobilized on Dynabeads M-280 Streptavidin (Invitrogen) essentially as described (59,60). The RNA was 5′ end labelled with [γ-^32^P] ATP. For EC assembly, RNAP was incubated with a hybrid of the DNA template strand annealed to the RNA in Elongation Buffer [EB: 20 mM HEPES (pH 7.6), 100 mM CH_3_CO_2_K (pH 7.6), 5 mM Mg(CH_3_COO)_2_, 0.1 mg BSA] for 5 min, then with the 3′end-biotinylated non-template DNA strand for 5 min and then with 2.5 g Heparine for 5 min at 20°C. Beads were subsequently washed three times with EB pre-heated at 50°C. Beads were resuspended in EB. For 1 nt RNA extension assays, 100 µM of NTP (ATP, 2'dATP or UTP) (NEB Biolabs) were added, and the mixture was incubated at 50°C. For the elongation and termination assays, 30 µM of NTPs mix were added. The mixture was incubated at 70°C for elongation assays and at 70, 80 or 90°C for termination assays. For TFS-induced cleavage assays, 150 nM TFS were added, and the mixture was incubated at 70°C. Reactions were stopped by transferring the sample into cold loading buffer containing formamid, and samples were heated to 95°C and analysed on a 28% polyacrylamid gel containing 7 M urea. For elongation assays and termination assays, the samples were analysed on a 15% polyacrylamid gel containing 7 M urea. The radioactively 5′-labelled RNA products were visualized with a FLA-5000 scanner (Fujifilm). Gel bands were quantified using Aida Image Analyzer (Raytest).

### Bead-based RNA intrinsic cleavage assays

For intrinsic RNA cleavage assays, ECs were assembled in Cleavage Buffer [CB: 20 mM HEPES (pH 9), 100 mM CH_3_CO_2_K (pH 9), 10 mM Mg(CH_3_COO)_2_, 0.1 mg BSA] and incubated at 70°C. The reactions were stopped by transferring the sample to ice. Beads were washed one time with CB and resuspended with CB with loading buffer containing formamid. Samples were heated to 95°C and analysed on a 28% polyacrylamid gel containing 7 M urea. The radioactively 5′-labelled RNA products were visualized with a FLA-5000 scanner (Fujifilm). Gel bands were quantified using Aida Image Analyzer (Raytest).

### Data analysis

The nucleotide (mis)incorporation rates obtained for various substrates and concentrations were fitted to the Michaelis–Menten equation, *k* = *k*_pol_ × [NTP]/(K_M_ + [NTP]), where *k*_pol_ is the maximum NTP incorporation rate of the enzyme, [NTP] is the substrate concentration and K_M_ is the Michaelis constant. Kinetic data (nucleotide addition, RNA hydrolysis) were fitted to a single exponential equation using non-linear regression in Sigmaplot (Systat Software Inc.). Discrimination ratios of complementary ATP (cATP) over c2′dATP and cATP over non-complementary UTP (ncUTP) were calculated by using the equation, (*k*_pol_,cNTP/K_M_,cNTP)/(*k*_pol_,2’dNTP/K_M_,2’dNTP) and (*k*_pol_,cNTP/K_M_,cNTP)/(*k*_pol_,ncNTP/K_M_,ncNTP), respectively.

## RESULTS

### TL is required for archaeal transcription

**Figure 1.**
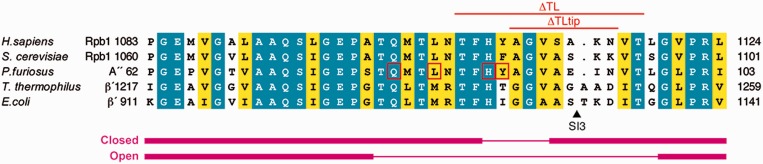
Sequence conservation of the TL in the three domains of life. Invariant (*blue*) and conserved (*yellow*) residues are shown in an alignment of TL sequences from human RNAP II (*Homo sapiens*), yeast RNAP II (*S. cerevisiae*), the archaeal RNAP (*P. furiosus)* and bacterial RNAPs (*T. thermophilus* and *E. coli*). Amino acid substitutions (by alanine, by serine for Y88) introduced for this study in subunit A'', and deletion mutations are indicated by red square and red lines, respectively. The triangle indicates the position of the insertion site of SI3 (188 aa) in the TL in *E. coli* RNAP. The α-helical and loop segments in the closed and open states of the TL are illustrated by thick and thin magenta lines, respectively.

We prepared six *Pfu* RNAP variants with TL mutations (Figure 1). TL mutations analysed include L83A, H87A and ΔTLtip (A'' A89-N95, *Sc* Rpb1 A1087-K1093), where the mobile tip of the TL is deleted. We also prepared a TL deletion mutant, ΔTL (A'' T85-T97, *Sc* Rpb1 T1083-T1095), where the size of the deletion was designed based on the structural information to prevent destabilization of the enzyme. In yeast, equivalent mutations were lethal (31,61). Furthermore, four supplementary mutants were analysed to clarify their participation in NTP over 2'dNTP discrimination: TL variants Q80A and Y88S, and A' R423A and A' N456A.

**Figure 2.**
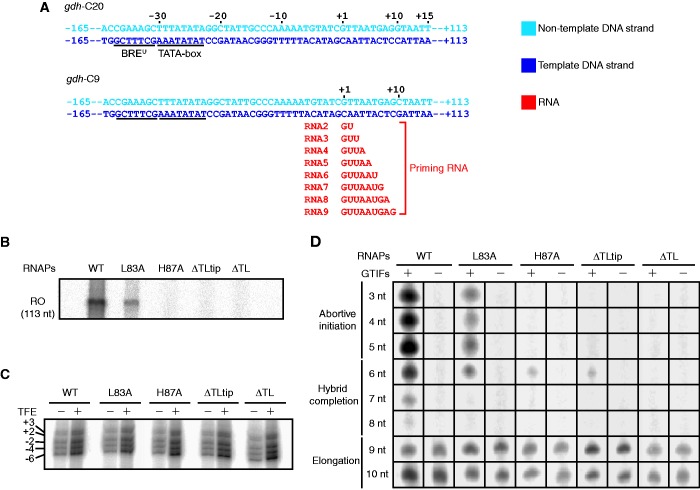
The TL is required for transcription initiation. (**A**) Schematic representation of initiation templates containing the strong *gdh* promoter of *P. furiosus*. The first C residue in the transcribed region of the *gdh*-C20 and *gdh*-C9 templates occurs at position +21 and +10, respectively (57). The sequence elements BRE^U^ and TATA-box are underlined. (**B**) Run-off (RO) *in vitro* transcription reactions were performed on the *gdh*-C20 template. (**C**) Permanganate footprinting analysis was performed on the *gdh*-C20 promoter in the presence or absence of TFE. (**D**) *In vitro* transcription reactions were performed on *gdh*-C9 template in the presence or absence of GTIFs and 1 nt extension products of priming RNAs RNA2 to RNA9 (panel A) were analysed on 28% polyacrylamide gels.

To analyse whether the TL RNAP mutants have an overall defect in promoter-dependent transcription, we subjected them to a promoter-specific transcription assay in which we used the strong *P.furiosus* glutamate dehydrogenase (*gdh*-C20) promoter in the presence of GTIFs (Figure 2A). The 113 nt run-off RNA products were synthesized by the wild-type (WT) RNAP and mutant L83A, demonstrating that L83 contributes to transcription, but is not essential (Figure 2B). In contrast, both TL deletion RNAP mutants and H87A displayed no transcription activity. These results show that the TL is critical for archaeal RNAP function, as for bacterial and eukaryotic RNAPs (22,31,62).

### TL is required for initiation

To investigate whether the initiation phase of transcription requires the TL, we carried out an abortive transcription assay, using closed and pre-opened versions of the *gdh* promoter template in combination with a dinucleotide GpU priming RNA (RNA2) that was complementary to the +1 and +2 positions in the template DNA strand (Supplementary Figure S1). By addition of the radioactive nucleotide [α-^32^P]-UTP, we tested whether mutant RNAPs could elongate the GpU RNA by 1 nt. The L83A variant synthesized the trinucleotide product but was less active than the WT enzyme (Supplementary Figure S1). The TL deletion mutants and H87A were not active.

The initiation defect of RNAP mutants was not largely due to a defect in DNA opening because similar results were obtained on DNA templates pre-opened by three mismatched nucleotides around the transcription start site (TSS, +1) or around the putative site of opening (−10). Transcription activity was also not increased by addition of TFE, which stimulates promoter opening and abortive transcription (Supplementary Figure S1) (4). Permanganate footprinting showed that mutations within the TL did not affect DNA opening, whereas TFE retains the stimulatory effect on all mutants (Figure 2C). Thus, the TL is not required for stable open complex formation or TFE binding during initiation complex formation but is important for the synthesis of the first nucleotide bonds.

### TL is required for initial RNA synthesis

We next analysed the capacity of WT RNAP and TL variants to extend a short RNA 3–9 nt in length by 1 nt in the presence or absence of GTIFs (Figure 2A). These initiation scaffolds mimicked progressive steps in transcription initiation. Assays with RNAs up to 5 nt mimic abortive initiation, whereas assays with 6–8 nt RNAs mimic hybrid completion (57,62). At an RNA length ∼11 nt, the bubble collapses and RNAP moves downstream (57).

The WT enzyme efficiently added 1 nt to short RNAs (3–6 nt) in these scaffolds, whereby the activity was dependent on GTIFs, and the synthesis of a 5 nt RNA was most effective (Figure 2D). On scaffolds mimicking hybrid completion, NTP incorporation activity decreased with RNA length and ceased at a length of 8 nt. However, addition of ATPs, GTP and UTP allowed elongation of RNA5 and RNA6 until RNA9 (Supplementary Figure S2). Therefore, the two successive RNA synthesis steps from RNA6 to RNA8 are necessary to maintain transcriptional activity, and they could not be obtained through a pre-synthesized RNA that binds the initiation complex. In contrast, extension activity is observed with RNA8 and RNA9 and was independent of GTIFs (Figure 2D). Except L83A, the TL mutant enzymes exhibited almost no activity, whereas all mutant RNAPs were able to add the complementary nucleotide on the RNA8 and RNA9 assemblies, independently from the presence of GTIFs. These results show that the TL including the mobile tip element and A'' H87 are required for transcription initiation.

### TL function in catalysis is universally conserved

**Figure 3.**
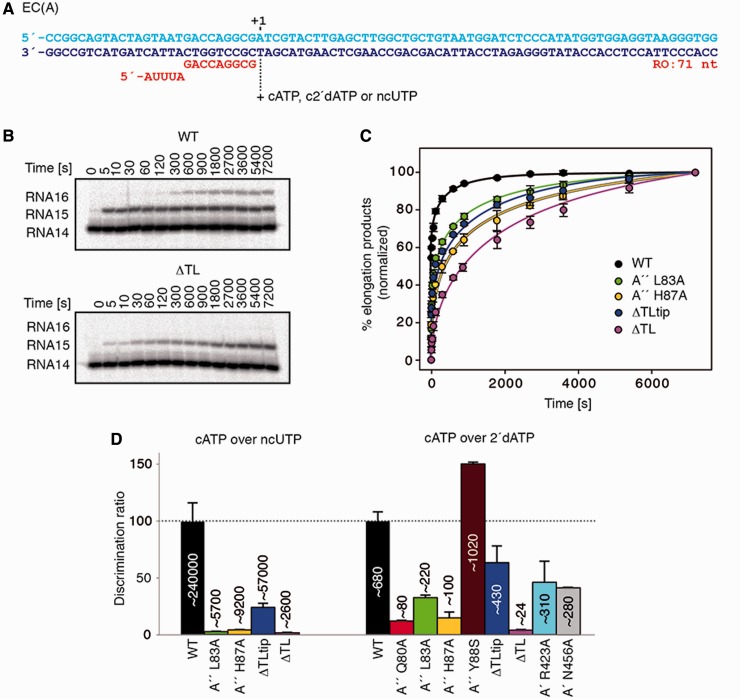
The role of the TL in catalysis and fidelity. (**A**) Elongation scaffold template EC(A) was used for nucleotide incorporation and misincorporation assays. The 5′ end of the RNA was labelled by ^32^P. cATP, complement ATP; ncUTP, non-complement UTP; c2'dATP, 2'complement deoxy-ATP. (**B**) Representative gels of cATP (100 μM) incorporation by WT and ΔTL RNAPs are shown. (**C**) Kinetics of cATP (100 µM) incorporation on the EC(A) template. Solid lines represent a single exponential fit of single nucleotide addition time course data. (**D**) Discrimination of cATP over c2'dATP and cATP over ncUTP by WT and mutants RNAPs were obtained by using the equation described in ‘Materials and Methods’ section. *k*_pol_ and K_M_ data are shown on Tables 1 and 2.

To explore the role of the archaeal TL during transcription elongation in more detail, we used an *in vitro* assembly of nucleic acids originally described by Kireeva *et al.* (59). Elongation scaffold EC(A) comprises a fully complementary 83 bp double-stranded DNA template and the radioactively labelled RNA14, which forms a 9 bp hybrid with the template DNA strand followed by a 5 nt 5′-overhang that is non-complementary to the DNA (Figure 3A). On this template, we determined the incorporation rate of a single cATP, 2’dATP, or a ncUTP. As the WT enzyme was fast in all assays, a lower than optimal reaction temperature was chosen that permitted determination of reaction kinetics. We measured *k*_pol_ and K_M_ values for nucleotide incorporation events on RNAP assembly on EC(A) and incubation with 2'dNTP at 50°C. The results of this test series are summarized in Tables 1 and 2.

**Table 1.** K_M_ and *k*_pol_ for incorporation and misincorporation reactions by WT and mutant RNAPs

RNAP	cATP		ncUTP		c2'dATP	
	*k*_pol_ (s^−1^)	K_M_ (µM)	*k*_pol_ (s^−1^)	K_M_ (µM)	*k*_pol_ (s^−1^)	K_M_ (µM)
WT	13 ± 0.6	29 ± 2	2.1 × 10^−3 ^± 0.4	1128 ± 92	1.5 × 10^−1 ^± 0.2	228 ± 20
A'' L83A	2.6 ± 0.2	53 ± 4	4.9 × 10^−3 ^± 0.5	566 ± 63	3.8 × 10^−2 ^± 0.4	170 ± 5
A'' H87A	1.1 ± 0.5	68 ± 5	1.3 × 10^−3 ^± 0.3	737 ± 50	2.5 × 10^−2 ^± 0.3	158 ± 16
ΔTLtip	2.1 ± 0.3	18 ± 1	1.6 × 10^−3 ^± 0.2	789 ± 60	5 × 10^−2 ^± 0.1	184 ± 8
ΔTL	4.2 × 10^−1 ^± 0.3	88 ± 10	7.4 × 10^−4 ^± 0.5	405 ± 40	1.9 × 10^−2 ^± 0.3	95 ± 5

*k*_pol_ (reaction rate at saturating NTP concentration) and K_M_ were obtained by hyperbolic fitting of the kinetic data into the Michaelis–Menten equation as described in ‘Materials and Methods’ section (Supplementary Figure S4). The values determined are shown with standard error (±).

**Table 2.** K_M_ and *k*_pol_ for cATP and c2'dATP incorporation reactions by mutant RNAPs

RNAP	cATP		c2'dATP	
	*k*_pol_ (s^−1^)	K_M_ (µM)	*k*_pol_ (s^−1^)	K_M_ (µM)
A'' Q80A	3.8 ± 0.4	29 ± 2	1.7 × 10^−1 ^± 0.1	116 ± 9
A'' Y88S	3.9 ± 0.3	26 ± 1	4.8 × 10^−2 ^± 0.5	325 ± 17
A' R423A	3.2 ± 0.2	30 ± 6	6.5 × 10^−2 ^± 0.6	188 ± 9
A' N456A	3.8 ± 0.4	31 ± 2	7 × 10^−2 ^± 0.4	161 ± 4

*k*_pol_ (reaction rate at saturating NTP concentration) and K_M_ were obtained as described in Table 1 and in ‘Materials and Methods’ section (Supplementary Figure S5).

Under our conditions, the WT enzyme added cATP at a *k*_pol_ of 13 s^-1^, similar to other RNAPs tested under *in vitro* conditions (22,31). The reaction with cATP was slower for all mutant RNAPs in comparison with the WT RNAP (Figure 3B and C, Supplementary Figures S3 and S4). Among all mutants tested, the ΔTL variant had the strongest effect on the incorporation rate, resulting in an ∼30-fold decrease, whereas the substrate affinity was decreased ∼3-fold. Thus, the archaeal TL is important, but not essential for NTP substrate binding and catalysis during elongation.

Among the single point mutations, H87A resulted in the strongest defect, decreasing *k*_pol_ by ∼10-fold and reducing substrate affinity by ∼2-fold (Table 1). The RNA synthesis rate with the L83A mutant decreased by 5-fold, whereas the substrate affinity was also reduced by ∼2-fold. Interestingly, deletion of the mobile tip of the TL reduced the incorporation rate while increasing the affinity for the substrate (Table 1). We conclude that the TL and specifically H87 are required for efficient catalysis, and that L83 and H87, but not the centrally located mobile tip of the TL, contribute to substrate binding.

### TL function in NTP selection and transcription fidelity

The misincorporation reaction of ncUTP advanced slowly, and *k*_pol_ was reduced by 3-4 orders of magnitude with all RNAPs, demonstrating the presence of an efficient discrimination mechanism for non-basepairing substrates in the archaeal enzyme (Table 1, Supplementary Figure S4). Although the reaction rates with ncUTP were similar with mutant and WT enzymes, the K_M_ values were lower for all TL mutants. Accordingly, the discrimination for ncUTP was relatively high at ∼240 000-fold for the WT RNAP, whereas the ability to recognize the correct NTP was reduced by ∼25-, 40- and 90-fold for the H87A, L83A and ΔTL mutant RNAPs, respectively (Figure 3D). Deletion of the TL tip resulted in only a moderate, 4-fold reduction on ncUTP discrimination. Thus, both L83 and H87 contribute to the recognition of the correct NTP, whereas the mobile tip of the TL is dispensable for this function.

### TL function in 2'dNTP discrimination

Incorporation and binding of 2'dATP by the WT enzyme was more efficient in comparison with the utilization of the ncUTP, as reflected by the lower, 680-fold discrimination (Figure 3D). As indicated in Table 1, the ΔTL RNAP mutant most poorly distinguished between ATP and 2'dATP, exhibiting a discrimination ratio that was reduced by almost 30-fold in comparison with the WT enzyme. The discrimination observed for the ΔTLtip mutant was only slightly reduced by ∼1.5-fold, whereas the single point mutations L83A reduced discrimination by 3-fold, and H87A by ∼7-fold.

These data indicated that the archaeal TL is important for 2'dNTP discrimination. We therefore analysed which TL residues contribute to 2’dNTP discrimination. Structural studies of yeast RNAP II delineated an interaction network between Rpb1 residues R446 (*Pfu* A' R423) and N479 (*Pfu* A' N456) with the 2'-OH group of the ribose moiety (19,24). In addition, a contact of the TL residue Q1078 (*Pfu* A'' Q80) with the 2’-OH group was observed recently (24). Functional studies on yeast RNAP II showed that serine substitution of yeast TL residue Rpb1 F1086 (*Pfu* A'' Y88) could improve the 2'dNTP discrimination (21).

These results prompted us to investigate the effect of analogous substitutions at homologues residues in the archaeal RNAP. In agreement with results from the eukaryotic system, mutation A'' Y88S resulted in a phenotype that improved discrimination of cATP over 2'dATP (Figure 3D, Table 2). Although the A' R423A and A' N456A substitutions had only a moderate effect on 2'dATP discrimination, we found that the A'' Q80A (yeast Q1078) substitution decreased the discrimination against 2'dATP by ∼8.5-fold (Figure 3D, Table 2, Supplementary Figure S5). These results show that a novel contact of a conserved glutamine residue in the TL with the NTP 2'-OH group may contribute to selection of NTP over 2'dNTP substrates.

### TL is not required for intrinsic RNA cleavage

**Figure 4.**
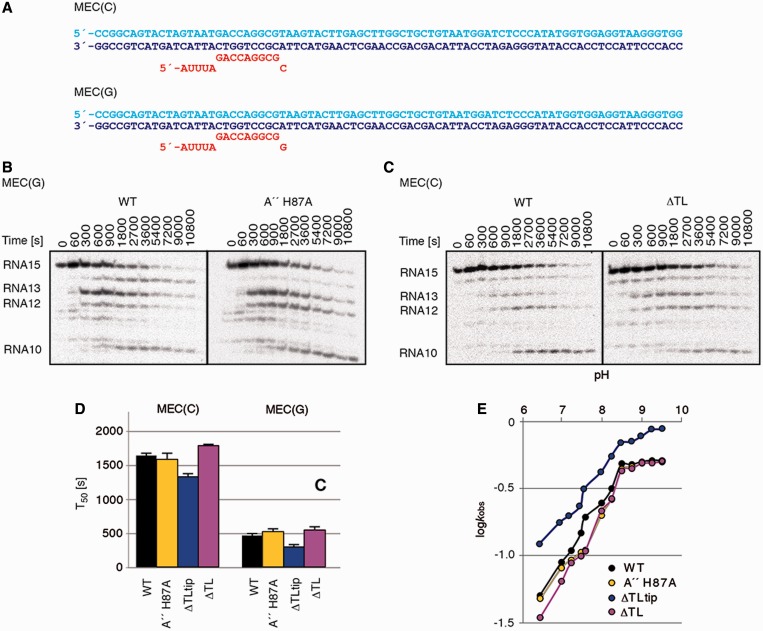
Intrinsic RNA cleavage does not require the TL. (**A**) Elongation scaffold templates used for intrinsic cleavage assays. MEC(C) and MEC(G) contained one mismatched cytidine and guanosine at the 3′end of RNA, respectively. (**B**) and (**C**) Representative gels of intrinsic phosphodiester bond hydrolysis in MEC(G) and MEC(C) by WT, H87A and ΔTL RNAPs. (**D**) The times required to reach 50% RNA cleavage products (T_50_) were obtained for MEC(C) and MEC(G) by single exponential fit of the intrinsic RNA cleavage product rate versus the reaction time (Supplementary Figure S7). (**E**) pH profiles of second phosphodiester bond hydrolysis on the MEC(G) scaffold by WT, A'' H87A, ΔTLtip and ΔTL RNAPs. The observed cleavage rates (*k*_obs_) were measured after a reaction time of 15 min.

To decipher the role of the archaeal TL during intrinsic RNA hydrolysis, we used the mismatched scaffold templates MEC(C) and MEC(G) in which the terminal nucleotide at the 3′-end (CMP or GMP) of RNA15 is non-complementary to the corresponding template DNA base, whereas the upstream residues of template and RNA strands in the 9-bp hybrid region were complementary (Figure 4A). Such complexes are thought to be in the pre-translocated state, where the mismatched nucleotide is in position +1, either mispaired with the DNA template strand or frayed away from the template (40,44). RNA dinucleotide cleavage requires backtracking of these complexes by 1 nt, referred to as +2-backtracked complexes (29,40,44).

Kinetic analysis of the intrinsic RNA cleavage reaction shows that neither the H87A mutation nor deletion mutants of the TL had a significant effect on the rate of RNA hydrolysis on either template in comparison with the WT activity (Figure 4B–D, Supplementary Figures S6 and S7). This agrees with one study in the bacterial system (63) but disagrees with another (46). The activity was higher on MEC(G) in comparison with MEC(C), with RNA degradation rates increased by ∼3-fold (Figure 4D), consistent with earlier findings that describe faster RNA cleavage on purine than on pyrimidine mismatches (40,46).

On MEC(C), the first cleavage product produced was RNA14, for both WT and mutant RNAPs (Figure 4C, Supplementary Figure S6). In contrast, with MEC(G), the first and major cleavage product is RNA13, whereas RNA14 was a minor cleavage product arising later during the course of the experiment. Only WT RNAP was able to produce the RNA14 cleavage product on the MEC(G) template, whereas cleavage with the H87A, ΔTLtip and ΔTL RNAPs yielded RNA13 and smaller cleavage products only (Figure 4B, Supplementary Figure S5). These results indicate that H87 and other residues in the TLtip region may influence the translocation state of the EC.

Recently, it was proposed that the TL of the *Thermus aquaticus* RNAP has a crucial role during intrinsic RNA cleavage activity at the penultimate phosphodiester bond by deprotonating the attacking water molecule with its histidine (β' H1242) (46). To test whether the related archaeal TL residue A'' H87 participates in intrinsic cleavage by this mechanism, we analysed the pH profiles of the dinucleotide hydrolysis reaction on the MEC(G) template with WT and mutant RNAPs. The inflection point at approximately pH 7.5 observed in the pH profile of the WT RNAP shifts to slightly higher pH in the H87A and the ΔTL mutant RNAPs, whereas the pH profiles of the ΔTLtip RNAP mutant closely resembles the WT profile (Figure 4E). Unlike for the bacterial system, the H87 mutant regained RNA cleavage activity up to WT levels at pH 9. Thus, H87 is not essential for RNA hydrolysis at the penultimate phosphodiester bond. Together, these data show that the TL is not required for intrinsic RNA cleavage by the archaeal RNAP.

### TL is not required for TFS-stimulated RNA cleavage

**Figure 5.**
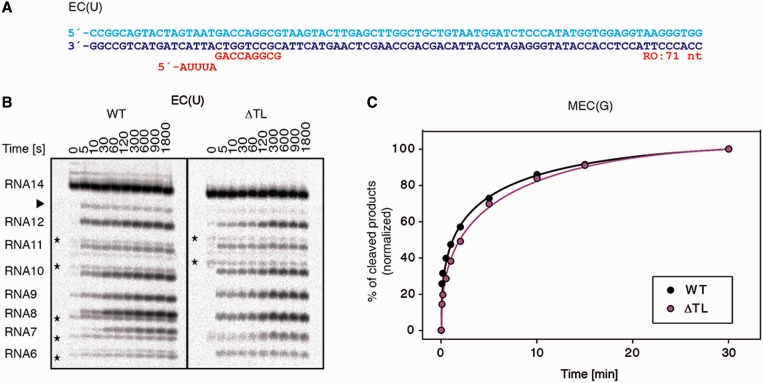
TL-independent TFS-induced RNA cleavage. (**A**) Elongation scaffold template EC(U) used for TFS-stimulated cleavage assays. The 5′ end of the RNA was labelled by ^32^P (**B**) Representative gels of TFS-induced phosphodiester bond hydrolysis on EC(U) scaffolds. Black asterisks indicate non-specific RNA degradation products. The arrow indicates the RNA products cleaved at the terminal 3′-phosphodiester bond. (**C**) Kinetics of the TFS-induced RNA cleavage reaction in MEC(G) scaffold template by WT and ΔTL RNAPs. Solid curves are the single exponential fits of the kinetics data. The reactions were performed in triplicates.

The RNA cleavage activity in the presence of TFS occurred with WT and mutant RNAPs faster than without TFS (Figure 5, Supplementary Figure S8). Moreover, TFS efficiently stimulated RNA hydrolysis in the absence of a 3′-mismatch (scaffold template EC(U), Figure 5A and B). In line with earlier results, the first cleavage occurs predominantly at the penultimate phosphodiester bond (Figure 5B, Supplementary Figure S8) (28,60). However, on EC(U), the WT enzyme was able to cleave also the terminal 3′-phosphodiester bond, but not the TL deletion mutants (Figure 5B, indicated by an arrow, Supplementary Figure S8), indicating that the TL affects the translocation state on matched templates also in the presence of TFS. The slight decrease in RNA cleavage activity noted with the ΔTL and ΔTLtip mutants (Figure 5C, Supplementary Figure S8) was also observed in the bacterial system and can be explained by a minor disarrangement of the active site caused by the deletion (47). Our results show that TFS-induced RNA cleavage can occur without participation of the TL.

### TL prevents aberrant transcription termination

**Figure 6.**
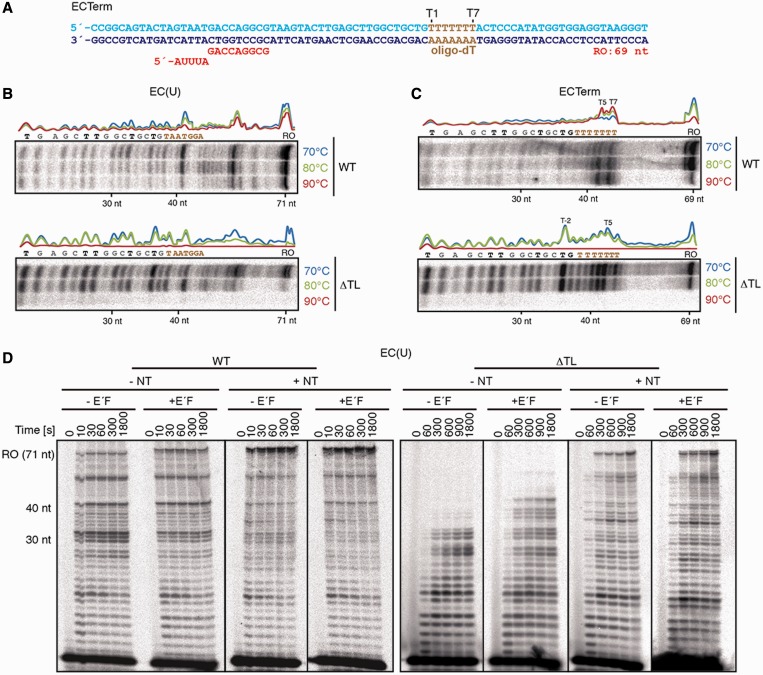
Implications of the TL in termination and processivity. (**A**) Elongation scaffold template ECTerm contains an oligo-dT sequence from positions +36 to +42. Transcription elongation assays with WT and ΔTL RNAPs were performed at 70, 80 and 90°C, with (**B**) EC(U) and (**C**) ECTerm templates. The curves are based on quantification of elongation products after a reaction time of 5 min for WT RNAP and 15 min for ΔTL RNAP (arbitrary units). (**D**) Transcription elongation kinetics with EC(U) at 70°C in the presence or absence of the non-template DNA strand (NT) and/or E'F subunits by WT and ΔTL RNAPs.

To investigate whether the TL plays a role during transcription termination, we compared the termination ability of WT and ΔTL RNAPs. Elongation assays were performed as described earlier in the text, but at increasing temperatures (70, 80 and 90°C) on template EC(U) and ECTerm, which contains a hepta-dT (T1 to T7) transcription termination sequence (Figure 6A). WT RNAP produced a run-off transcript under all conditions tested (Figure 6B and C). Processivity improved with increasing temperatures, apparent as fewer and less pronounced premature stop sites at dT. On both EC(U) and ECTerm templates, the overall amount of transcript slightly decreased at higher temperatures, whereas on the ECTerm template, the termination efficiency improved (Figure 6B and C) as reported (49,51). Termination with WT RNAP occurred mainly at T5 and T7, whereas the ΔTL RNAP terminated transcription primarily at T-2, indicating that the TL contributes to anti-termination at T-rich sequences. At 90°C, the ΔTL enzyme was unable to elongate the scaffold RNA by >8 nt. At 70–85°C, premature stops were more prevalent with the TL deletion mutant in comparison with the WT enzyme (Supplementary Figure S9). This effect was not reduced by increased NTP concentration (Supplementary Figure S9).

Previously, the non-template DNA strand (NT) and RNAP subunits E' and F (homologues of eukaryotic Rpb7 and Rpb4, respectively) were shown to be required for full processivity (64). We therefore reconstituted WT and ΔTL enzymes lacking E'F and added either E'F, the NT or both during otherwise standard EC assemblies. We found that processivity of the ΔTL enzyme was highly diminished in the absence of both NT and E'F so that elongation ceased at an RNA length of 30–35 nt, whereas the WT RNAP was still able to produce a 71 nt run-off transcript under all conditions (Figure 6D). Addition of E'F allowed the ΔTL enzyme to slightly extend RNA synthesis until a length of ∼35–40 nt. The run-off product was produced by the ΔTL RNAP only in the presence of the NT. However, neither WT nor mutant RNAPs were responsive to E'F addition in the presence of the NT (Figure 6D, Supplementary Figure S9). We conclude that the ΔTL RNAP mutant exhibits a temperature-sensitive processivity defect and is prone to termination at T-rich sequences. These results show that the TL is required for suppressing termination at non-terminator sites.

## DISCUSSION

**Figure 7.**
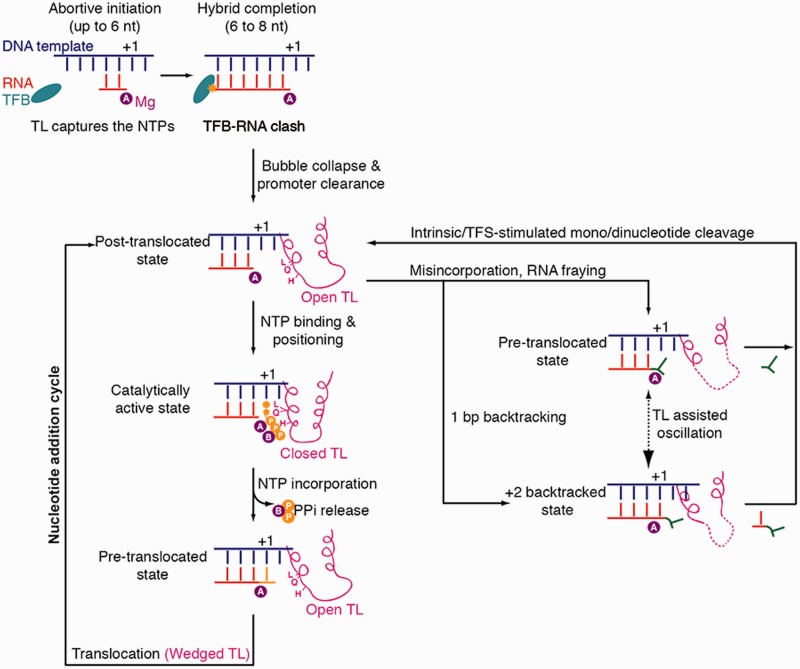
Schematic representations of TL dynamics in distinct transcription phases. The TL is essential for initial synthesis until a complete DNA-RNA hybrid formation (8 nt). The TL is important in transcription fidelity and catalysis during transcription elongation phase. Although L83 and H87 contribute to the recognition of the correct NTP, Q80 contributes to the recognition of the 2′OH-group of the NTP. When misincorporation occurs, the TL influences translocation but does not contribute in intrinsic or factor-stimulated RNA cleavage during proofreading.

Although previous TL analysis concentrated on transcription elongation, we report here functions of the TL during transcription initiation and termination (Figure 7). The archaeal TL was absolutely required for initiation, whereas it was not essential for elongation. The initiation function of the TL is apparently catalytic because RNAP variants were fully responsive to TFE, TFS and E'F, and transcription bubble formation was normal. For catalysis in initially transcribing complexes, active site closure and synthesis stimulation by the TL were crucial, as A'' H87 and the TLtip region were essential for initiation. It was suggested that RNA extension beyond 6 nt causes RNA separation from DNA to redirect nascent RNA to its exit channel (62). This is consistent with our finding that RNA6 and RNA7 were weakly extended by RNAP. Thus, the TL is essential for capturing NTPs during initial transcription before a stable DNA–RNA hybrid is present in the active centre, and this TL function is more important than its elongation function.

Our results also provided insights into TL function during the discrimination of NTPs from 2'dNTPs. Mutagenesis of residues that were proposed to interact with the NTP 2’-OH group showed that A' R423 and N456 moderately affected 2'dNTP discrimination, whereas residue A'' Q80 was more important, consistent with its proposed role to couple 2'-OH recognition with TL closure (23). Consistent with previous reports of bacterial and eukaryotic systems (31,32), A'' H87 was important for discrimination of NTPs against 2'dNTPs. Taken together, this indicates that TL closure requires interactions of H87 with the NTP triphosphate and of Q80 with the 2'OH group of the NTP, and that these interactions are important for nucleotide incorporation fidelity.

Transcription fidelity also relies on proofreading, a post-incorporation mechanism that involves cleavage of a dinucleotide from the RNA 3′-end containing the misincorporated nucleotide. In our assays, dinucleotide cleavage was preferred over mononucleotide cleavage, consistent with a catalytic role of the RNA 3′-end (44). The TL was not required for dinucleotide cleavage, but some RNAPs with mutant TLs were defective in mononucleotide cleavage. This indicated that the TL influences translocation, consistent with the TL being part of a Brownian ratchet that underlies translocation and swings against the nascent base pair of the hybrid to test its stability (65). Proofreading is stimulated by extrinsic RNA cleavage factors such as TFS, and we found that the TL is also dispensable for TFS-stimulated cleavage, consistent with structures illustrating that the TL is in the locked conformation in the presence of the eukaryotic TFS counterpart, TFIIS (29,30).

Finally, our results revealed a function of the TL in transcription termination. We show that the sensitivity of the archaeal RNAP to poly-T sequences is increased on TL truncation, showing that the TL prevents aberrant termination at non-terminator sites, to ensure processive RNA synthesis. In the absence of the TL or on its mutation, RNAP apparently is more prone to pausing and termination at T-rich sites.

## SUPPLEMENTARY DATA

Supplementary Data are available at NAR Online: Supplementary Figures 1–9 and Supplementary Methods.

## FUNDING

The Deutsche Forschungsgemeinschaft [SFB646, SFB960, TR5, GraKo1721, CIPSM, NIM, an Advanced Grant of the European Research Council, the LMUinnovativ project Bioimaging Network, the Jung-Stiftung, and the Vallee Foundation]. Funding for open access charge: DFG [SFB 960].

*Conflict of interest statement*. None declared.

## Supplementary Material

Supplementary Data
