# Supplementary material for: The RNA polymerase trigger loop functions in all three phases of the transcription cycle
Source: Nucleic Acids Res. 2013 May 21;41(14):7048–59. doi: 10.1093/nar/gkt433 (PMC3737540; doi:10.1093/nar/gkt433)
Supplement: Supplementary Data [file supp_41_14_7048__index.html]

The RNA polymerase trigger loop functions in all three phases of the transcription cycle — The RNA polymerase trigger loop functions in all three phases of the transcription cycle — Supplementary Data 

# The RNA polymerase trigger loop functions in all three phases of the transcription cycle

## Supplementary Data

files

**Files in this Data Supplement:**

- Supplementary Data - pdf file
