# Supplementary material for: The RNA polymerase trigger loop functions in all three phases of the transcription cycle
Source: Nucleic Acids Res. 2013 May 21;41(14):7048–59. doi: 10.1093/nar/gkt433 (PMC3737540; doi:10.1093/nar/gkt433)
Supplement: Supplementary Data [file supp_gkt433_nar-01061-v-2013-File011.pdf]

SUPPLEMENTAL INFORMATION

Supplemental Materials and Methods

In vitro transcription assays with pre-opened templates

Open templates contained mismatches at position -9, -10 and -11 (“open upstream”) or -2, -1 and +1 (“open start”) relative to the transcription start site in *gdh*-C20 and were prepared as reported (7). Transcription assay was carried out as described in *Materials and Methods.* RNA was analyzed by electrophoresis in 28% polyacrylamide/urea gels.

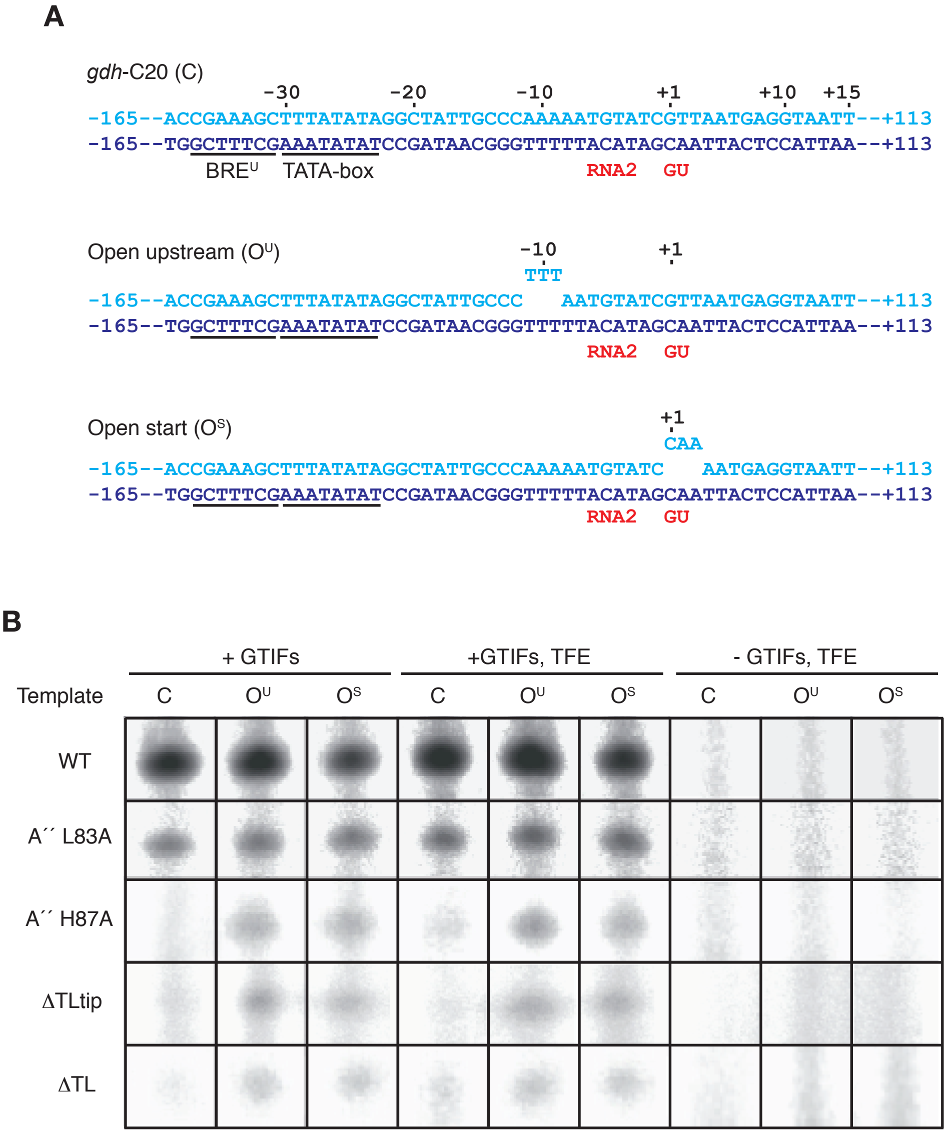

Figure S1.
(**A**) Closed and pre-opened templates containing the gdh promoter. The BRE^U and the TATA-box are underlined. The *gdh*-C20 template is described in Figure 1. The templates "open upstream" and "open start" comprised a mismatch region from position –11 to -9 and +1 to +3 relative to the transcription start site, respectively. (**B**) In vitro transcription reactions were performed on closed (C), "open upstream" (O^U) and "open start" (O^S) *gdh*-C20 templates in the presence (+) or absence (-) of GTIFs and TFE. The trinucleotide product after extension of RNA2 by 1 nt was detected.

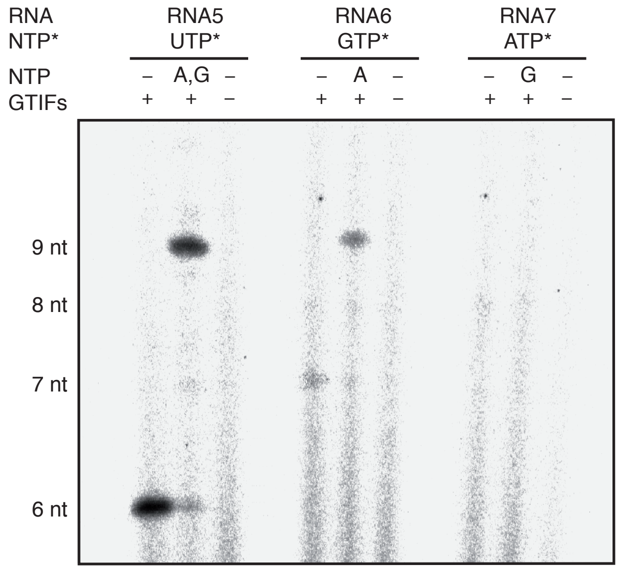

Figure S2.

Two successive RNA synthesis steps from RNA6 to RNA8 are necessary for hybrid completion. In vitro transcription reactions were performed on *gdh*-C9 template with WT RNAP in the presence or absence of GTIFs and extension products of priming RNAs, RNA5 to RNA7 (Figure 2A), were analyzed on a 28% polyacrylamid gel containing 7M urea.

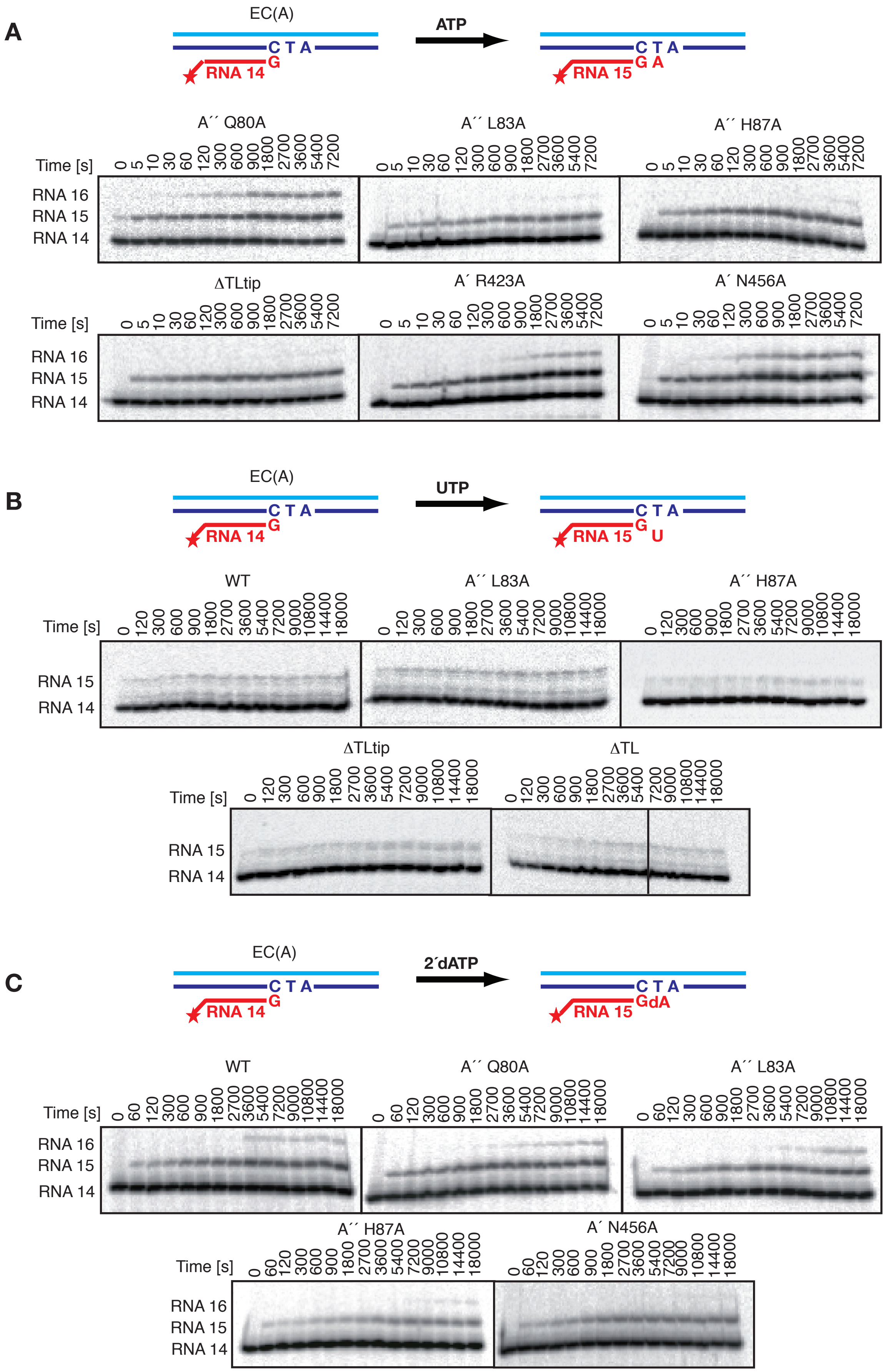
A
EC(A)
ATP
C T A
G
RNA 14
RNA 15
G A
A´´ Q80A
A´´ L83A
A´´ H87A
ΔTLtip
A´ R423A
A´ N456A
Time [s]
RNA 16
RNA 15
RNA 14
B
UTP
U
WT
ΔTL
C
2´dATP
GdA

Figure S3

Figure S3.

Representative gels of (**A**) cATP (100 µM) incorporation, (**B**) ncUTP (1 mM) misincorporation and (**C**) c2'dATP (100 µM) incorporation by WT and mutant RNAPs are shown, respectively. Schematic representation above the gel pictures describes the (mis)incorporation of (**A**) cATP, (**B**) ncUTP or (**C**) c2'dATP on the EC(A) scaffold template. The red asterisk indicates that the RNA is ^{32}P-labeled at the 5' end.

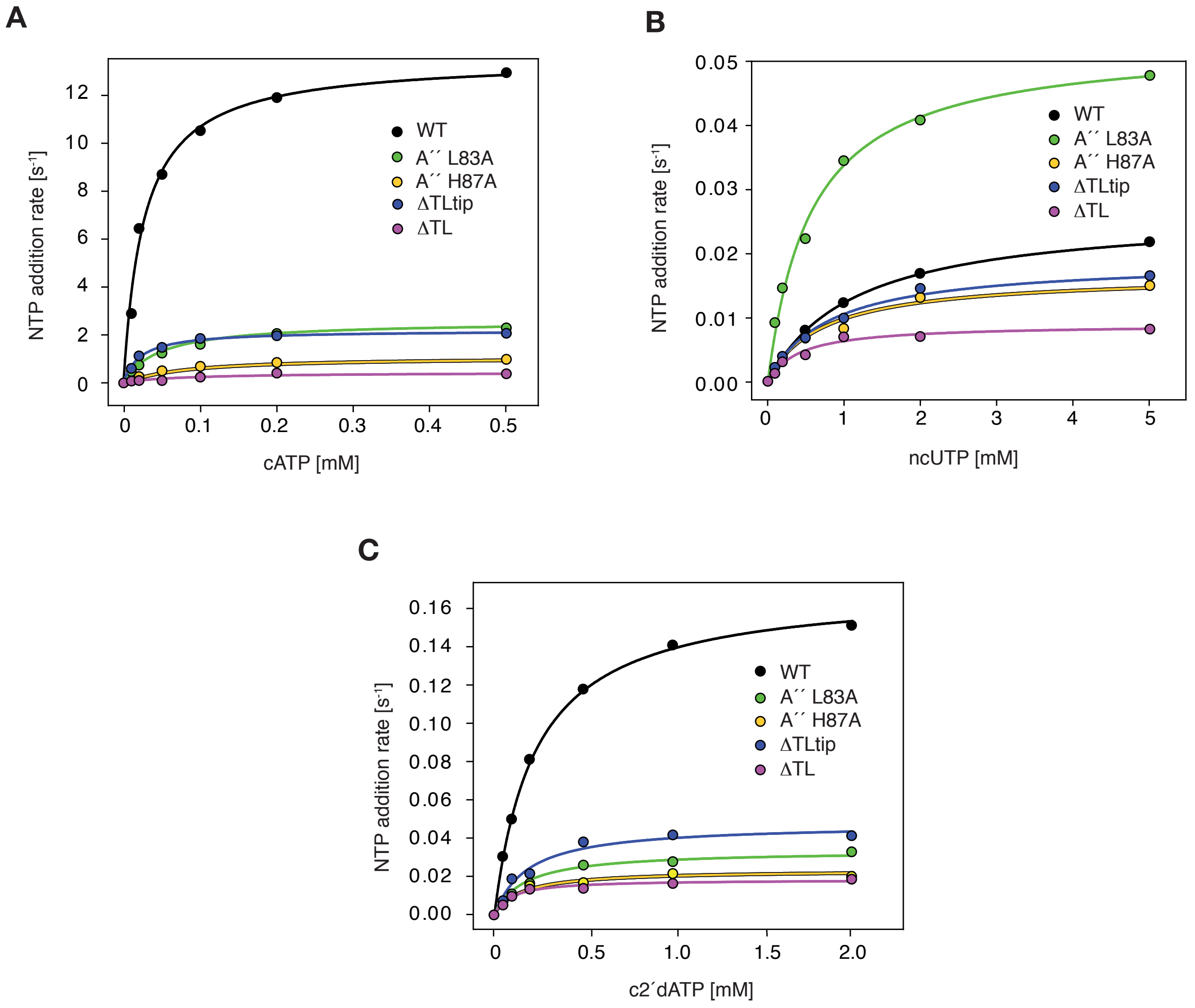

Figure S4.
(Mis)incorporation dependence on the concentrations of (**A**) cATP, (**B**) ncUTP or (**C**) c2'dATP by WT, A'' L83A, A'' H87A, ΔTLtip and ΔTL RNAPs. The scaffold template EC(A) was incubated with the indicated substrate concentrations at 50°C. The reactions at each NTP concentration were performed in triplicates. The curves show the data fit into the Michaelis-Menten equation described in *Materials and Methods*.

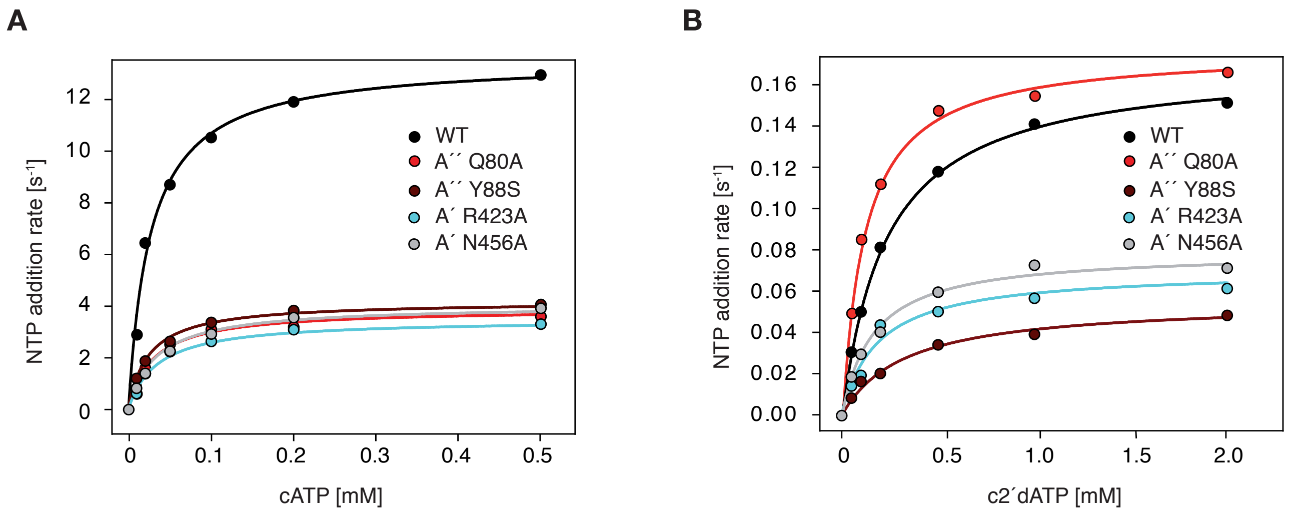

Figure S5.
Incorporation dependence on concentrations of (**A**) cATP and (**B**) c2'dATP by WT, A'' Q80A, A'' Y88S, A' R423A and A'' N456A RNAPs. The scaffold template EC(A) was incubated with the indicated substrate concentrations at 50°C. The reactions at each NTP concentration were performed in triplicates. The curves show the data fit into the Michaelis-Menten equation described in *Materials and Methods*.

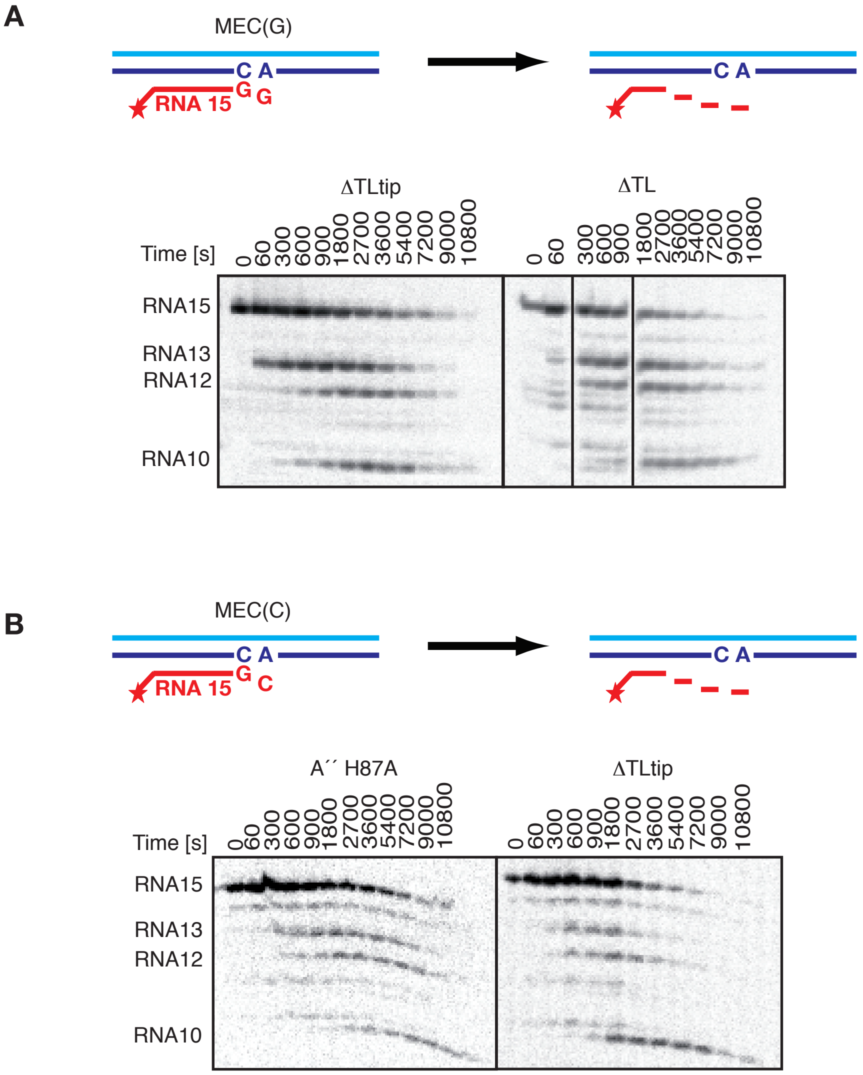

Figure S6.
Representative gels of intrinsic phosphodiester bond hydrolysis in (**A**) MEC(G) and (**B**) MEC(C) and scaffold templates by mutant RNAPs. Schematic representation above the gel pictures describes the reactions. The red asterisk indicates that the RNA is P-labeled at the 5' end.

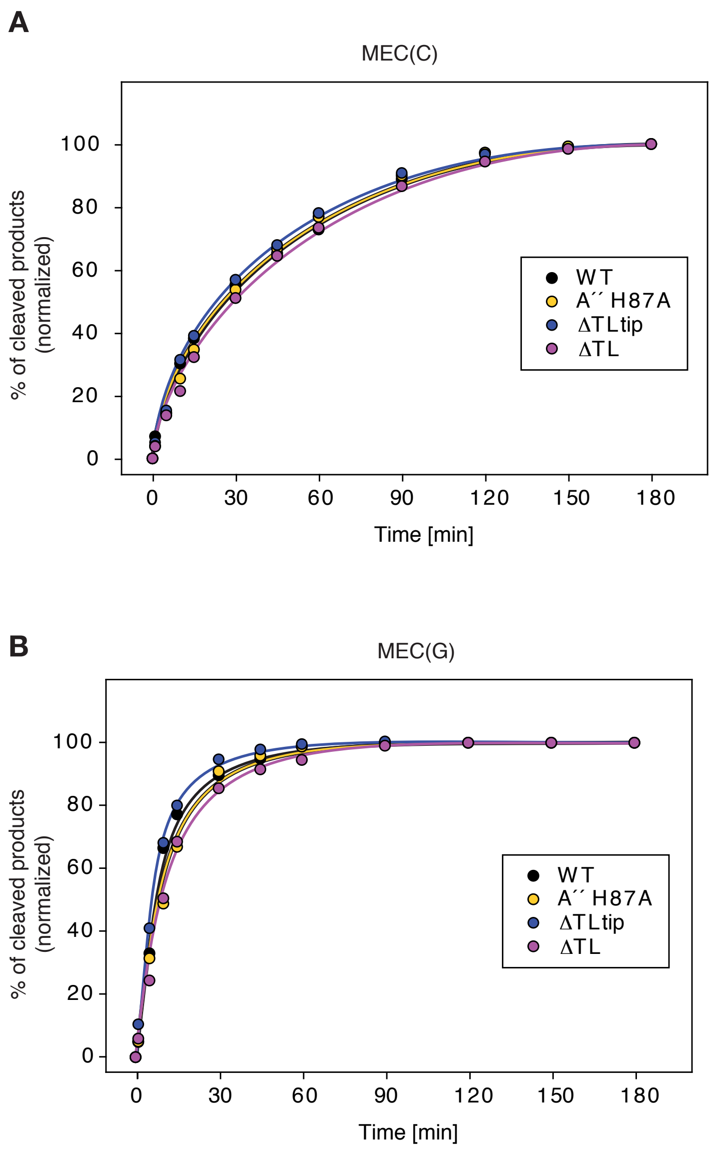

Figure S7.

Kinetics of the intrinsic RNA cleavage reaction in (**A**) MEC(G) and (**B**) MEC(C) scaffold templates by WT, A'' H87A, ΔTLtip and ΔTL RNAPs. Solid curves are the single exponential fits of the kinetics data. The reactions were performed in triplicates.

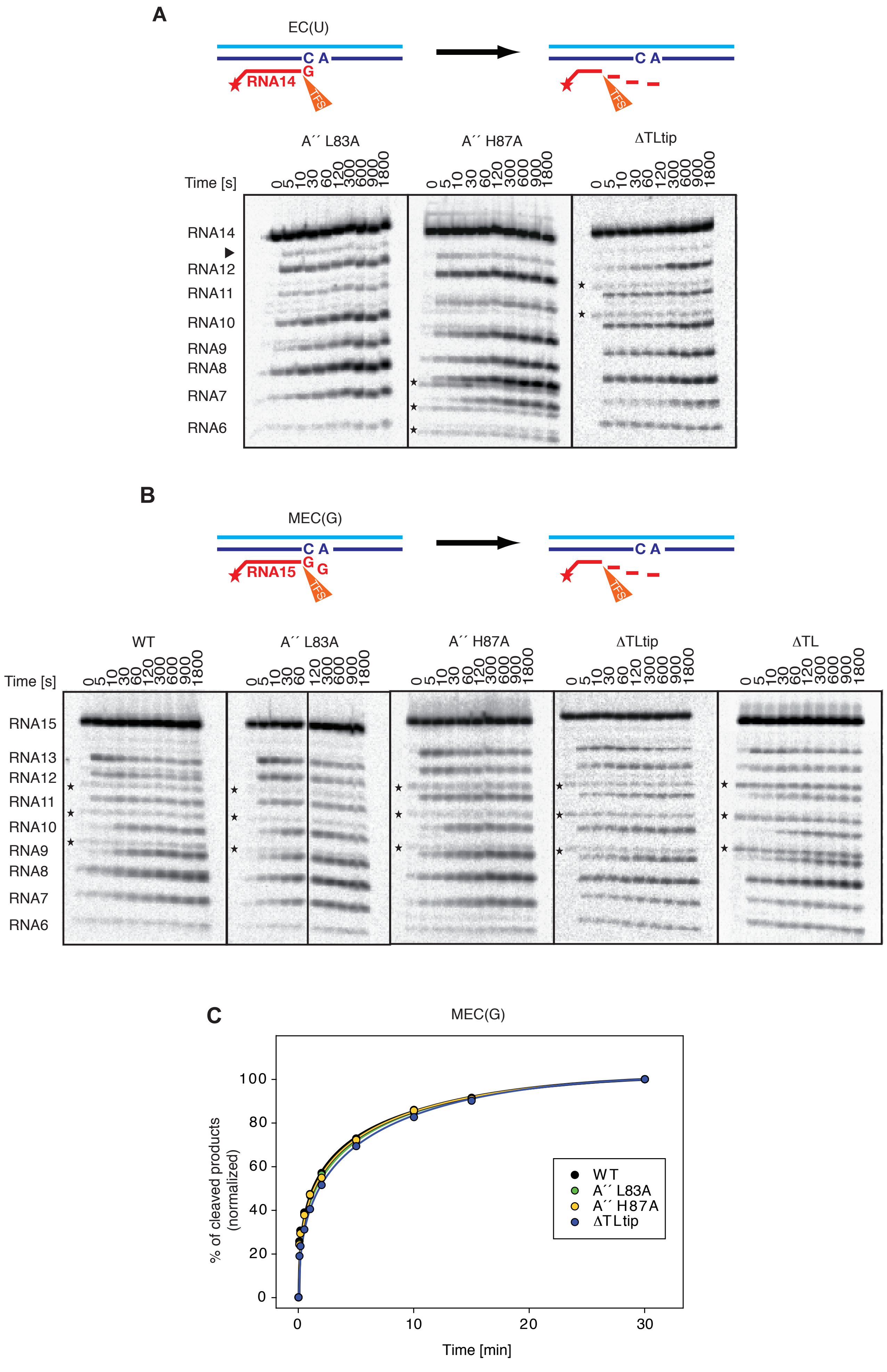

A
EC(U)
C A
G
RNA14
TFS
C A
TFS
A´´ L83A
A´´ H87A
ΔTLtip
Time [s]
0 5 10 30 60 120 300 600 900 1800
RNA14
RNA12
RNA11
RNA10
RNA9
RNA8
RNA7
RNA6
B
MEC(G)
C A
G G
RNA15
TFS
C A
TFS
WT
A´´ L83A
A´´ H87A
ΔTLtip
ΔTL
Time [s]
0 5 10 30 60 120 300 600 900 1800
RNA15
RNA13
RNA12
RNA11
RNA10
RNA9
RNA8
RNA7
RNA6
C
MEC(G)
% of cleaved products (normalized)
0
20
40
60
80
100
0
10
20
30
Time [min]
WT
A´´ L83A
A´´ H87A
ΔTLtip

Figure S8

Figure S8.

Representative gels of TFS induced phosphodiester bond hydrolysis in (**A**) EC(U) and (**B**) MEC(G) scaffold templates by WT and mutant RNAPs are shown. Black asterisks indicate nonspecific RNA degradation products. Schematic representation above the gel pictures describes the reactions. The red asterisk indicates that the RNA is ^{32}P-labeled at the 5' end. The arrow indicates the RNA products cleaved at the terminal 3'-phosphodiester bond. (**C**) Kinetics of the TFS induced RNA cleavage reaction in MEC(G) scaffold template by WT, A'' L83A, A'' H87A, and ΔTLtip RNAPs. Solid curves are the single exponential fits of the kinetics data. The reactions were performed in triplicates.

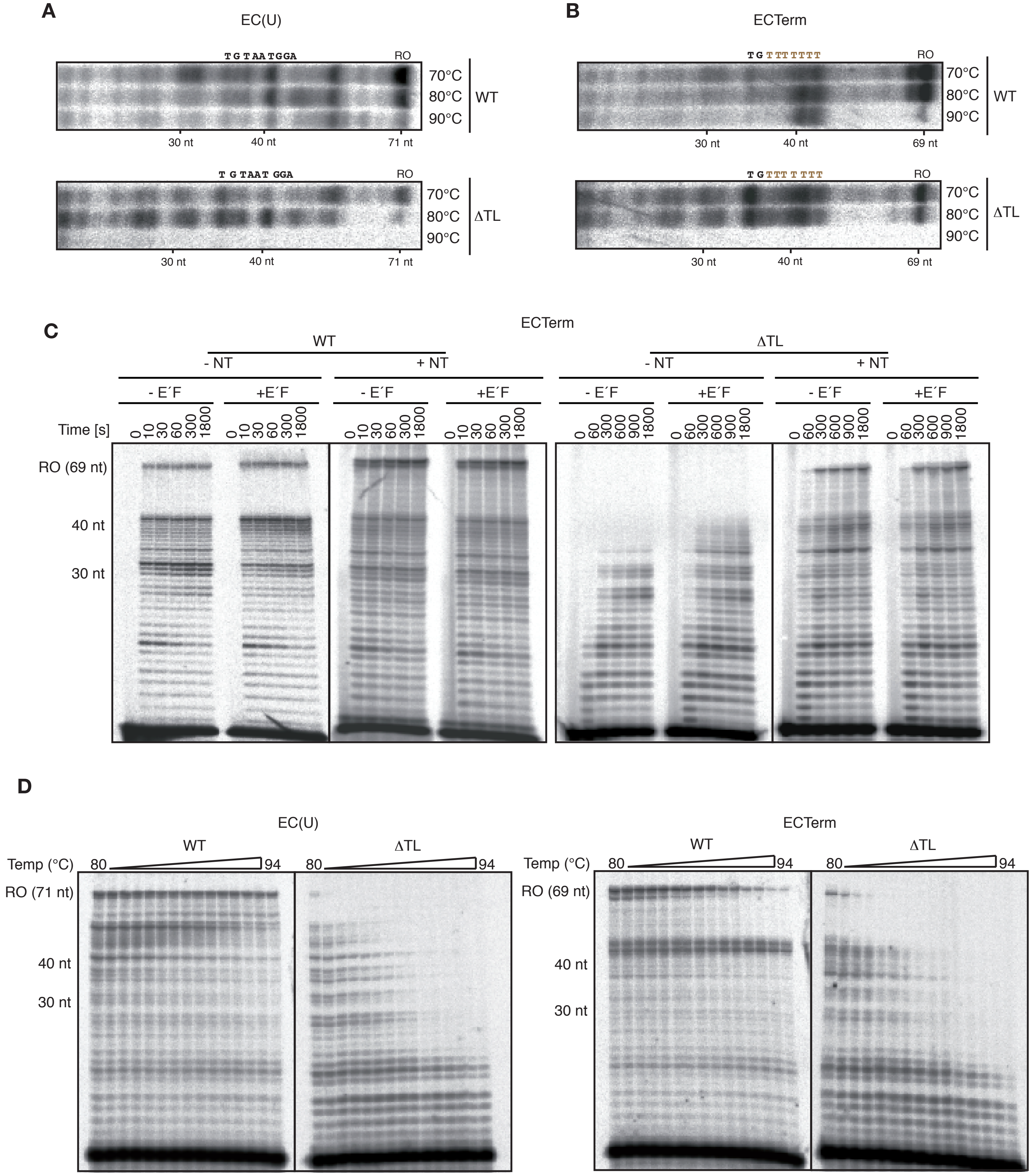
A
EC(U)
T G T A A T G G A
RO
70°C
80°C
90°C
WT
30 nt
40 nt
71 nt
T G T A A T G G A
RO
70°C
80°C
90°C
ΔTL
30 nt
40 nt
71 nt
B
ECTerm
T G T T T T T T T
RO
70°C
80°C
90°C
WT
30 nt
40 nt
69 nt
T G T T T T T T T
RO
70°C
80°C
90°C
ΔTL
30 nt
40 nt
69 nt
C
ECTerm
WT
ΔTL
- NT
+ NT
- E´F
+E´F
Time [s]
0 10 30 60 300 1800
0 60 300 600 900 1800
RO (69 nt)
40 nt
30 nt
D
EC(U)
ECTerm
WT
ΔTL
Temp (°C)
80
94
RO (71 nt)
RO (69 nt)
40 nt
30 nt

Figure S9

Figure S9.

Transcription elongation assays by WT and ΔTL RNAPs were performed at 70, 80 and 90°C, with (**A**) EC(U) and (**B**) ECTerm templates, in the presence of 100 μM NTPs. Samples were incubated for 5 min with WT RNAP and for 15 min with ΔTL RNAP. (**C**) Transcription elongation kinetics on the ECTerm scaffold template in the presence (+) or absence (-) of the non-template DNA strand (NT) and/or E'F subunits with WT and ΔTL RNAPs. (**D**) Transcription elongation was assayed between 80 to 94°C in 1°C increments on EC(U) and ECTerm scaffold templates. Samples were incubated for 5 min with WT RNAP and for 15 min with ΔTL RNAP.
